# Hydrogel Bioinks of Alginate and Curcumin-Loaded Cellulose Ester-Based Particles for the Biofabrication of Drug-Releasing Living Tissue Analogs

**DOI:** 10.1021/acsami.3c07077

**Published:** 2023-08-16

**Authors:** João P. F. Carvalho, Maria C. Teixeira, Nicole S. Lameirinhas, Filipe S. Matos, Jorge L. Luís, Liliana Pires, Helena Oliveira, Martinho Oliveira, Armando J. D. Silvestre, Carla Vilela, Carmen S. R. Freire

**Affiliations:** † CICECO−Aveiro Institute of Materials, Department of Chemistry, University of Aveiro, Aveiro 3810-193, Portugal; § CICECO−Aveiro Institute of Materials, EMaRT Group - Emerging: Materials, Research, Technology, School of Design, Management and Production Technologies Northern Aveiro, University of Aveiro, Oliveira de Azeméis 3720-511, Portugal; ‡ Department of Biology & CESAM, University of Aveiro, Aveiro 3810-193, Portugal

**Keywords:** 3D bioprinting, composite hydrogel bioinks, cellulose ester-based particles, drug delivery, living tissue analogs

## Abstract

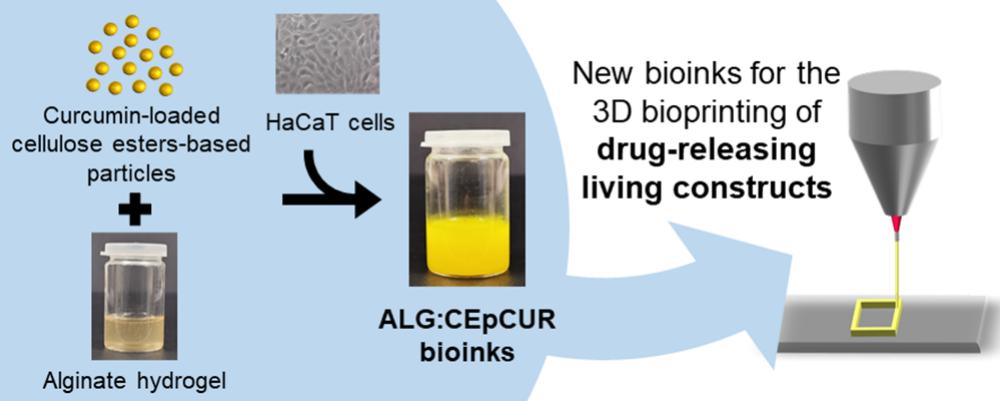

3D bioprinting is a versatile technique that allows the fabrication of living tissue analogs through the layer-by-layer deposition of cell-laden biomaterials, viz. bioinks. In this work, composite alginate hydrogel-based bioinks reinforced with curcumin-loaded particles of cellulose esters (CEpCUR) and laden with human keratinocytes (HaCaT) are developed. The addition of the CEpCUR particles, with sizes of 740 ± 147 nm, improves the rheological properties of the inks, increasing their shear stress and viscosity, while preserving the recovery rate and the mechanical and viscoelastic properties of the resulting fully cross-linked hydrogels. Moreover, the presence of these particles reduces the degradation rate of the hydrogels from 26.3 ± 0.8% (ALG) to 18.7 ± 1.3% (ALG:CEpCUR_10%) after 3 days in the culture medium. The 3D structures printed with the ALG:CEpCUR inks reveal increased printing definition and the ability to release curcumin (with nearly 70% of cumulative release after 24 h in PBS). After being laden with HaCaT cells (1.2 × 10^6^ cells mL^–1^), the ALG:CEpCUR bioinks can be successfully 3D bioprinted, and the obtained living constructs show good dimensional stability and high cell viabilities at 7 days post-bioprinting (nearly 90%), confirming their great potential for application in fields like wound healing.

## 1 Introduction

Additive manufacturing has caught the interest of many scientific domains in recent years, and three-dimensional (3D) bioprinting, its biomedical counterpart, has granted the field of tissue engineering with a panoply of new opportunities by allowing the fabrication of complex 3D living tissue analogs.^1^ This technique consists in the computer-controlled layer-by-layer deposition of cell-laden biomaterials, the so-called bioinks, and allows to create living constructs with tailored size and morphology.^2^ Understandably, the bioink is a key element for the success of the 3D bioprinting process, and the demand for new and improved bioinks is growing. Bioinks must be carefully designed to (i) possess the physical and mechanical features required for bioprinting (e.g., adequate rheological properties) and (ii) grant the conditions for cells to survive the entire procedure and to prosper in the bioprinted construct.^3^ Considering their intrinsic characteristics and composition, bioinks are often divided into two major families: scaffold-free (i.e., tissue strands, cellular pellets, and tissue spheroids) or scaffold-based options (viz. hydrogel-based bioinks, microcarrier-based bioinks, and decellularized extracellular matrix (dECM)-based bioinks).^4^

Hydrogels are the most investigated class of bioinks, and they are frequently studied in extrusion-based techniques, where the application of mechanical or pneumatic pressure causes the extrusion of the bioink through a nozzle.^5^ Hydrogels are polymeric networks that resemble the cellular microenvironment, and may be easily obtained by the cross-linking of synthetic^6^ or natural polymers^7,8^ or their derivatives (e.g., alginate,^9^ gelatin,^10^ and chitosan^11^). Among natural polymers, alginate is one of the most renowned choices for 3D bioprinting applications.^7^ This anionic polysaccharide, composed of glucuronate (G) and mannuronate (M) units, has been explored in numerous works for the bioprinting of different tissues (i.e., skin,^12^ liver,^13^ bone,^14^ or cartilage^15^) given its simple cross-linking mechanism with divalent cations (e.g., Ca^2+^) and known biocompatibility.^9^ However, there are a few setbacks to the use of this biopolymer in hydrogel-based bioinks for 3D bioprinting. Alginate-based hydrogels do not possess cell adhesion moieties and often have weak mechanical properties and uncertain degradation rates.^16^ Given this, alginate is commonly combined with different materials to originate new hydrogel-based bioinks with enhanced properties.^8,17^ In this topic, the combination with other biopolymers^17,18^ (e.g., cellulose,^19^ chitosan,^20^ or gelatin^21^) or the reinforcement with nanostructures (e.g., nanoparticles,^22−24^ nanocrystals,^25^ and nanofibers^21,26^) is particularly relevant. The development of composite alginate hydrogel-based bioinks is a research field with great potential, with several publications exploring the use of different particles (e.g., hydroxyapatite,^22^ silica,^23^ or polydopamine^24^), usually to improve the mechanical or rheological properties of the hydrogels or their biological performance, or to grant the inks with new functionalities (e.g., magnetic properties and conductivity).^27,28^

Cellulose is another natural polymer with great potential for biological applications, with well-known biocompatibility and versatility for chemical modification.^29^ Cellulose esters (e.g., cellulose acetate and cellulose nitrate) are one of the most important families of cellulose derivatives.^30,31^ For example, cellulose acetate is obtained by the acetylation of cellulose, and has found applications in textiles, packaging, and biomedical fields, being frequently used in the shape of electrospun fibers, membranes, and (nano)particles.^32,33^ However, the use of cellulose acetate in the development of cell-laden bioinks for 3D bioprinting has not yet been described. Cellulose nitrate (also known as nitrocellulose) is obtained through nitration of cellulose, and it has also found applications in biomedical domains, mostly as a material for membranes and biosensors.^33−35^ Nonetheless, and although the work reported by Li et al.^36^ highlighted the potential of this cellulose derivative to be used in scaffolds for the proliferation of several cell lines, the application of cellulose nitrate in the formulation of bioinks for 3D bioprinting is also still unexplored.

In this perspective, the present work describes the development of composite alginate hydrogel-based bioinks loaded with cellulose ester-based particles for the 3D bioprinting of skin cells. This work innovates by exploiting spherical particles, based on cellulose esters, as a vehicle for drugs or bioactive compounds and also as an additive to produce alginate hydrogel-based bioinks with improved properties. Thus, the cellulose ester-based particles were loaded with a model-drug, viz. curcumin (CUR), which is a lipophilic compound extracted from the roots of *Curcuma longa*, with known antioxidant and anti-inflammatory potential, and a distinctive yellow color.^37^ The use of curcumin in drug-delivery is often jeopardized by its very low solubility in water, and therefore, its incorporation in spherical carriers is a valuable strategy to overcome such limitations, as demonstrated by Zamboni et *al.* in a study combining curcumin-loaded nanoparticles of poly(lactic acid) with alginate/gelatin hydrogels for *in situ* immunoregulation. However, the authors did not explore the 3D bioprinting of cell-laden hydrogels.^38^ In fact, the development of cell-laden composite bioinks containing drug carriers to originate 3D bioprinted living constructs with drug-delivery abilities is an almost untouched topic, with no previous work describing the use of cellulose-based particles for such applications. Therefore, the present work constitutes a relevant step in the development of multifunctional bioinks aimed at generating 3D bioprinted structures with different functionalities (i.e., drug-delivery). The thorough characterization of the cellulose ester-based particles, composite inks, resulting fully cross-linked hydrogels, and 3D-bioprinted constructs, confirms the potential of these bioink formulations to create 3D living tissue analogs with drug delivery capabilities for application in the biomedical field.

## 2 Materials and Methods

### 2.1 Chemicals and Materials

Sodium alginate from brown algae (low viscosity, 4–10 cP), calcium chloride anhydrous (≥98%), and phosphate buffer saline (PBS, pH 7.4) were purchased from Sigma-Aldrich (Sintra, Portugal). Sample of mixed cellulose esters (cellulose acetate, DS = 0.16, and cellulose nitrate, DS = 1.33, as determined by Fourier transform infrared-attenuated total reflection (FTIR-ATR) spectroscopy^39^) were obtained from FILTER-LAB (Barcelona, Spain). Acetone (≥99.5%) was obtained from Honeywell (Charlotte, MA, USA), and curcumin (95%) was supplied by Alfa Aesar (Kandel, Germany). Ultrapure water (Type 1, 18.2 MΩ cm at 25 °C) was obtained via a Simplicity Water Purification System (Merck, Darmstadt, Germany).

Dulbecco’s modified Eagle’s medium (DMEM) was purchased from PAN-Biotech (Germany), and 3-(4,5-dimethylthiazolyl-2)-2,5-diphenyltetrazolium bromide (MTT, 98%) was supplied by Alfa Aesar (Kandel, Germany), while dimethyl sulfoxide (DMSO, ≥ 99.9%) and trypsin-EDTA solution (0.25% trypsin, 0.02% EDTA) were purchased from Sigma-Aldrich. Fetal bovine serum (FBS) and fungizone were obtained from Gibco (Life Technologies, Carlsbad, CA, USA). l-Glutamine solution (200 mM) and penicillin/streptomycin solution (100×) were purchased from Grisp (Porto, Portugal). The LIVE/DEAD cell viability assay kit was supplied by Sigma-Aldrich (Sintra, Portugal). The HaCaT cell line was purchased from Cell Lines Services (Eppelheim, Germany).

### 2.2 Preparation of Cellulose Ester-Based Particles

The preparation of cellulose ester particles (CEp) was achieved by a water-on-polymer method.^40^ Briefly, 80.0 mg of the mixed cellulose esters was dissolved in 20.0 mL of acetone. Ultrapure water was then added to this solution at a flow rate of 0.4 mL min^–1^ using a Harvard Apparatus PHD Ultra syringe pump (Holliston, Massachusetts, EUA), equipped with a 0.45 mm needle gauge and under magnetic stirring (500 rpm). The resulting cellulose ester particles were centrifuged posteriorly and washed twice with ultrapure water.

### 2.3 Preparation of Curcumin-Loaded Particles

The preparation of curcumin (CUR) loaded CEp particles (CEpCUR) was achieved using the same method described for the blank counterparts (CEp). However, given the photosensitivity of curcumin, this process was performed in the dark. Thus, 80.0 mg of cellulose esters was dissolved in 20.0 mL of a solution of curcumin (0.2 mg mL^–1^) in acetone, and ultrapure water was similarly added at 0.4 mL min^–1^ flow rate through a 0.45 mm needle, at 500 rpm agitation. CEpCUR were centrifuged, washed twice with ultrapure water, protected from light, and stored in the refrigerator.

The incorporation percentage of curcumin in the particles was assessed via ultraviolet–visible (UV–vis) spectroscopy (Thermo Scientific Evolution UV-vis 600, Thermo Fisher Scientific). Specifically, the determination of the remaining curcumin in solution after the production of CEpCUR (supernatant) was calculated by measuring the absorbance at 430 nm.^41,42^ The concentration of curcumin in the supernatant was calculated using the following calibration curve Absorbance = 0.0687 × Concentration + 0.0181, (*R*^2^ = 0.9995).

### 2.4 Preparation of the Composite Alginate Hydrogel-Based Inks and of the Fully Cross-Linked Hydrogels

The alginate (ALG) hydrogel-based inks were obtained by combining an aqueous solution of 4% (w/v) of ALG, and different contents of CEp or CEpCUR (1, 5, and 10 wt % relative to the mass of alginate), as described in Table 1. Therefore, for a total volume of 10.0 mL of ink, 400.0 mg of ALG, and different amounts of CEp/CEpCUR were mixed in 8.0 mL of ultrapure water. The formulations were pre-cross-linked by the careful addition of 2.0 mL of a 0.5% (w/v) CaCl_2_ solution in order to modulate their rheological properties, as already described in other works.^43,44^ The obtained inks were left to stabilize overnight in the refrigerator.

**Table 1:** Composition of the Different Hydrogel-Based Inks of ALG, CEp, and CEpCUR

**Sample**	**ALG [mg]**	**CEp [mg]**	**CEpCUR [mg]**	**Water [mL]**	**CaCl**_**2**_**0.5% (w/v) [mL]**
ALG:CEp_1%	400.0	4.0	-	8.0	2.0
ALG:CEp_5%	400.0	20.0	-	8.0	2.0
ALG:CEp_10%	400.0	40.0	-	8.0	2.0
ALG:CEpCUR_1%	400.0	-	4.0	8.0	2.0
ALG:CEpCUR_5%	400.0	-	20.0	8.0	2.0
ALG:CEpCUR_10%	400.0	-	40.0	8.0	2.0

The fully cross-linked hydrogels tested along this work were obtained from the composite inks by immersion overnight in a 2% (w/v) aqueous solution of CaCl_2_, allowing the alginate hydrogel to be completely cross-linked.

### 2.5 Fourier Transform Infrared-Attenuated Total Reflection (FTIR-ATR) Spectroscopy

The FTIR-ATR spectra of cellulose esters, curcumin, CEp and CEpCUR were obtained with a PerkinElmer FT-IR System Spectrum BX spectrophotometer (PerkinElmer Inc., Waltham, MA, USA), using a single horizontal Golden Gate ATR cell (Specac, London, UK), in the range of 600–4000 cm^–1^ and with a resolution of 4 cm^–1^ over 32 scans.

### 2.6 Scanning Electron Microscopy (SEM)

The observation of CEp, CEpCUR, and the 3D printed constructs was performed using a HR-FESEM SU-70 Hitachi microscope (Hitachi High-Technologies Corporation, Tokyo, Japan) operating at 4 kV. 3D printed grid-like structures were previously freeze-dried for 48 h and then placed in a SEM stub using conductive carbon adhesive tape. In the case of CEp and CEpCUR particles, a drop of each suspension was placed over the carbon tape and left to dry overnight. All samples were coated with a carbon layer using an EMITECH K950 coating system prior to SEM observation. The particle size determination was performed with ImageJ software by the analysis of a minimum of 300 particles in each SEM micrograph.

### 2.7 In Vitro Cytotoxicity Evaluation

The cytotoxic impact of CEp, CEpCUR particles, and of the fully cross-linked hydrogels was evaluated against HaCaT (human keratinocyte) cells for periods of 24, 48, or 72 h, using the MTT assay.^45^ Cells were cultivated at 37 °C in a 5% CO_2_ humidified atmosphere, using DMEM supplemented with 10% FBS, 250 μg mL^–1^ fungizone, 10000 U mL^–1^ penicillin/streptomycin, and 2.0 mM l-glutamine. Daily observation of the cells was performed in an Eclipse TS100 microscope (Nikon, Tokyo, Japan).

In the case of CEp and CEpCUR, different amounts of particles were resuspended in DMEM to obtain the same concentrations as in the inks formulations, viz., 0.4 mg mL^–1^ for ALG:CEp_1% and ALG:CEpCUR_1%; 2 mg mL^–1^ for ALG:CEp_5% and ALG:CEpCUR_5%; and 4 mg mL^–1^ for ALG:CEp_10% and ALG:CEpCUR_10%. On the other hand, the fully cross-linked hydrogels obtained from ALG, ALG:CEp, and ALG:CEpCUR ink formulations were incubated in DMEM at 37 °C with 5% CO_2_ for 24 h to prepare the extracts. In both cases, six wells of HaCaT cells were treated identically but exposed simply to DMEM to serve as controls.

For each test, wells on a 96-well plate were seeded with 6000 cells/well (for 24 h), 4000 cells/well (48 h), or 2000 cells/well (72 h) and incubated for 24 h. Then, culture medium was replaced by 100 μL of each sample/extract to test, and cells were incubated further for 24, 48, or 72 h hours. After this exposure time, 50 μL of MTT (1.0 g L^–1^) were added to each well, and the plate was further incubated for 4 h. Subsequently, the medium was replaced with 150 μL of DMSO, and the plate was placed in an orbital shaker for 2 h. The absorbance of the samples was assessed using a BioTek Synergy HT plate reader (Synergy HT Multi-Mode, BioTeK, Winooski, VT) at 570 nm with blank corrections. The cell viability was calculated using the following formula, in which Abs_sample_ is the absorbance of the sample, Abs_DMSO_ is the absorbance of DMSO, and Abs_control_ is the absorbance of the control:
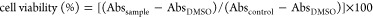
1

### 2.8 Rheological Characterization

The rheologic evaluation of the inks and the respective fully cross-linked hydrogels was performed using a Kinexus Lab+ Rheometer (Malvern Instruments Limited, Malvern, United Kingdom) equipped with a cone–plate geometry (angle of 4° and 40 mm diameter). Rotational tests of the inks were made with a gap of 1 mm, and in a shear rate range of 0.1–100 s^–1^, while oscillatory tests were performed at a frequency of 1 Hz and a shear strain range of 0–100%, using cylinder-shaped (15 mm diameter, 5 mm height) samples of the fully cross-linked hydrogels.

The recovery rate of the inks was evaluated with a 3-step oscillatory test: (i) evaluation of *G*′ in relaxation, at 1 Pa for 1 min; (ii) measurement of *G*′ under 100 Pa for 10 s; and (iii) a second evaluation of the *G*′ in relaxation, at 1 Pa for 1 min. The recovery (%) was calculated as
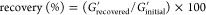
2where *G*′_Initial_ corresponds to the average *G*′ in the first relaxation phase and *G*′_Recovered_ corresponds to the *G*′ measured in the second relaxation phase. All measurements were performed at 20 °C.

### 2.9 Mechanical Compression Tests

The assessment of the mechanical properties of the fully cross-linked hydrogels was performed via mechanical compression tests, using cylindrical samples (15 mm diameter and 5 mm height). These tests were performed in a uniaxial Instron 5966 machine (Instron Corporation, USA) in compression mode, with a static load cell of 500 N, and at a speed of 5 mm min^–1^. All essays were performed until 80% of strain was reached, and Young’s Modulus and compressive stress were calculated using the Bluehill 3 Software (Version 3.22, Illinois Tool Works Inc., Glenview, IL, USA).

### 2.10 Degradation Assays

To evaluate the degradation rate of the fully cross-linked hydrogels of ALG, ALG:CEp, and ALG:CEpCUR, cylindrical samples (15 mm diameter and 5 mm height) were weighed and placed in 2.0 mL of the testing media (DMEM or PBS) for 3 days at 37 °C. At selected time points, samples were withdrawn from the media, their excess media was removed, and the hydrogels were weighed again. Then, the degradation rate was calculated by the following equation:
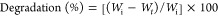
3where *W*_i_ is the initial weight and *W_t_* is the weight of the sample at each time point.

### 2.11 3D Printing Assays

The 3D printing assays were performed using a 3D-Bioplotter printer (Developer Series, EnvisionTEC GMBH, Gladbeck, Germany). First, the printing parameters (printing pressure and printing speed) were optimized using the ALG, ALG:CEp, and ALG:CEpCUR samples by printing straight filaments with 750 mm length using the 0.25 mm (inner diameter) nozzle, at varying printing speeds (5–15 mm s^–1^) and pressures (0.5–2.0 bar). Posteriorly, CAD software was used to design grid-like structures with dimensions of 20 × 20 mm, with a single layer height of 0.320 mm and a spacing of 2.25 mm between filaments to be printed with the inks.

Grid-like structures with different number of layers were 3D printed using the ALG, ALG:CEp and ALG:CEpCUR inks at 20 °C. After that, the printed constructs were fully cross-linked by immersion in a 2% (w/v) aqueous solution of CaCl_2_ for 15 min. The printability (*Pr*) of the hydrogel-based inks was evaluated from the SEM micrographs of 2-layered constructs, with ImageJ software, using the following equation:^24,46^
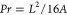
4where *L* is the perimeter and *A* is the area of the pores of the bilayered constructs.

### 2.12 Drug-Release Studies

In order to investigate the release of curcumin from the 3D-printed constructs, grid-like structures (20 × 20 mm) obtained by printing the ink formulations containing curcumin (ALG:CEpCUR_1%, ALG:CEpCUR_5%, and ALG:CEpCUR_10% inks) were immersed in 30.0 mL of PBS at 37 °C, for 24 h under moderate agitation. Aliquots (2.0 mL) of the media were collected at defined time points and replaced by the same volume of fresh medium, previously heated at 37 °C. The cumulative release of curcumin into the media was assessed by UV–vis spectroscopy (Thermo Scientific Evolution UV–vis 600, Thermo Fisher Scientific) at a wavelength of 430 nm.^47^ The percentage of cumulative release was calculated using the formula:^48,49^
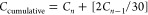
5where *C*_*n*_ and *C*_*n*–1_ are the concentrations of curcumin in solution at times *n* and *n*–1.

### 2.13 3D Bioprinting Using HaCaT Cells and LIVE/DEAD Assay

HaCaT cells were incorporated into the ALG, ALG:CEp_10% and ALG:CEpCUR_10% hydrogel-based inks to prepare the bioinks for 3D bioprinting assays. To achieve this, the hydrogel-based bioinks were prepared following the previously described methodology, but in a sterile environment, in a laminar flow chamber. All the reagents and materials used in the formulations were previously sterilized via at least 3 cycles of 20 min of UV irradiation. HaCaT cells were centrifuged and resuspended in 1 mL of DMEM, and homogeneously mixed with the formulations prior to the pre-cross-linking step to achieve a final cell density of 1.20 × 10^6^ cells mL^–1^. Bioinks were then pre-cross-linked and transferred into the printer cartridge.

Regarding the 3D bioprinting process itself, grid-like structures with 20 × 20 mm and an inner spacing of 2.25 mm were bioprinted using the cell-laden ALG, ALG:CEp_10% and ALG:CEpCUR_10% bioinks, using a printing pressure of 2 bar and a printing speed of 10 mm s^–1^, with a nozzle of 0.25 mm inner diameter. The final 3D bioprinted grid-like structures were fully cross-linked using a CaCl_2_ 2% (w/v) solution for 15 min and then incubated in DMEM for 7 days.

Cell viabilities after 1, 3, and 7 days post-bioprinting were evaluated using the LIVE/DEAD assay (propidium iodide/calcein AM). Following the specifications of the manufacturer, the fluorescent dyes were prepared and added to the structures for 30 min at 37 °C. The samples were then observed via confocal microscopy (Zeiss LCM 880, Carl Zeiss, Oberkochen, Germany), and the percentage of viable cells was calculated using the equation:
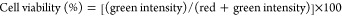
6

### 2.14 Statistical Analysis

The statistical analysis was made with the analysis of variance (ANOVA) and Tukey’s test (OriginPro, version 9.0.0, OriginLab Corporation, Northampton, MA, USA) with statistical significance defined at *p* < 0.05.

## 3 Results and Discussion

**Figure 1:**
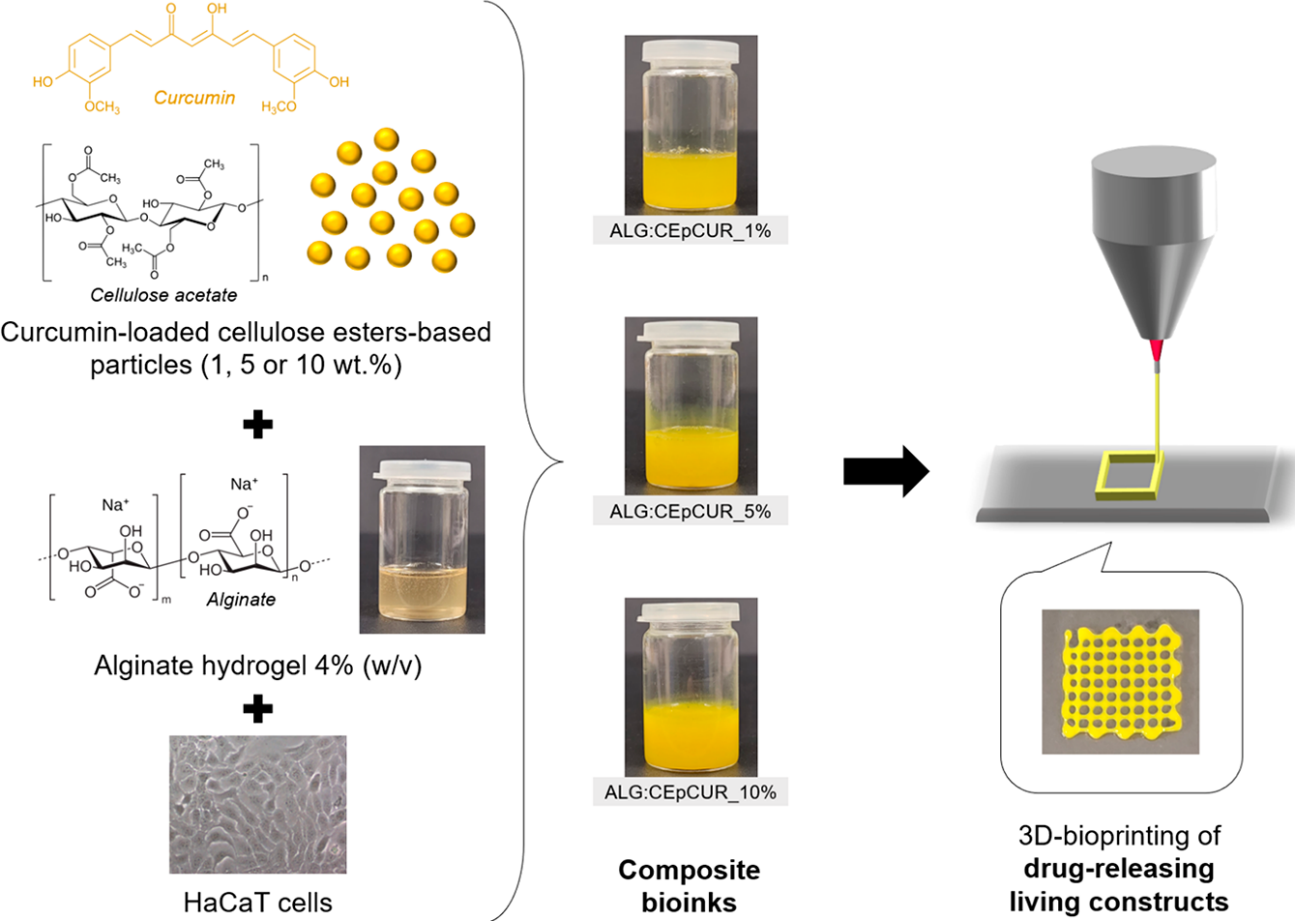
Schematic representation of the procedure used to prepare the composite bioinks based on alginate, curcumin-loaded cellulose ester-based particles, and skin cells for the 3D bioprinting of drug-releasing structures.

The present study describes the development of composite bioinks obtained by the incorporation of curcumin-loaded cellulose ester particles into alginate-based hydrogels for the 3D bioprinting of HaCaT human keratinocyte cells (Figure 1). First, the curcumin-loaded particles were prepared and characterized in terms of their morphology, structure, size, and *in vitro* cytotoxicity. Then, distinct ink formulations were obtained by the combination of alginate (4% (w/v)) with different contents of particles (1, 5, and 10 wt %, in respect to the mass of alginate). All the prepared inks were evaluated regarding their rheological features, and the corresponding fully cross-linked hydrogels were characterized in terms of their mechanical and viscoelastic properties, *in vitro* cytotoxicity, and degradability in different media. Next, the 3D printing parameters for the new inks were optimized, and their printability was evaluated. The drug-release capability of the resulting constructs was evaluated. Finally, the bioprinting of cell-laden 3D structures with the bioinks containing HaCaT cells was investigated via the LIVE/DEAD assay, up to 7 days of post-bioprinting.

### 3.1 Characterization of the Cellulose Ester-Based Particles

**Figure 2:**
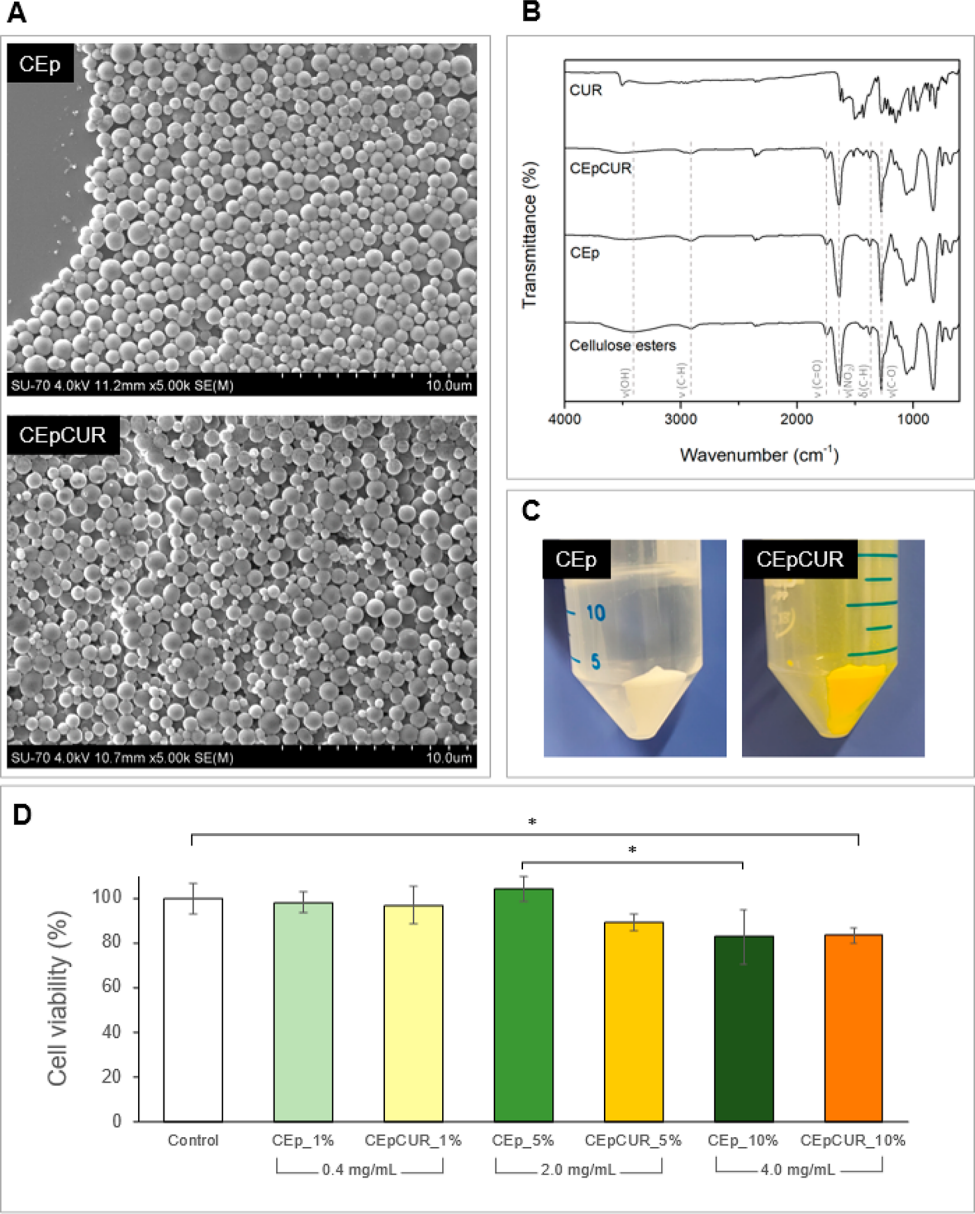
(A) Scanning electron micrographs of CEp and CEpCUR particles; (B) FTIR–ATR spectra of cellulose esters, CEp, CEpCUR, and CUR; (C) photographs of the CEp and CEpCUR particles after centrifugation; (D) evaluation of the cell viability of HaCaT cells after 24 h of exposure to CEp or CEpCUR particles. Values are presented as the mean of six replicates (*p* < 0.05: *).

The first step in the preparation of the alginate composite bioinks was the production of cellulose ester-based particles (CEp) with suitable size and morphology via dissolution of the cellulose derivatives in acetone followed by regeneration in water. The scanning electron microscopy (SEM) micrographs of the CEp (Figure 2A) confirmed the production of spherical and individualized particles with a smooth surface. These particles presented an average diameter of 738 ± 139 nm, which is in line with previous data concerning other particles based on cellulose esters produced under similar conditions.^40,50^ Moreover, the SEM observation of the curcumin (CUR) loaded particles (CEpCUR), produced by following the same approach, showed that CEpCUR are equally spherical, smooth, and well dispersed and with sizes of 740 ± 147 nm (Figure 2A), confirming that the incorporation of curcumin does not impact to a great extent the morphology or the dimensions of the particles. Given the selected bioprinting nozzle (0.25 mm), the size of these particles is deemed adequate for the forthcoming 3D bioprinting process.

The Fourier transform infrared–attenuated total reflection (FTIR-ATR) spectroscopic analysis (Figure 2B) of the cellulose esters, curcumin, and both CEp and CEpCUR proves that their production process did not affect the structure of the cellulose derivatives. In fact, the spectra of both types of particles are quite similar to the spectra of the mixed cellulose acetate and cellulose nitrate, with the main vibrations at 3416 cm^–1^ (−OH stretching), 2918 cm^–1^ (symmetric C–H stretching), 1740 cm^–1^ (C=O of the acetate groups), 1633 cm^–1^ (−NO_2_ stretching), 1371 cm^–1^ (C–H bending of the CH_3_ in the acetyl group), and 1275 cm^–1^ (C–O stretching). Additionally, the emergence of a small peak at 1513 cm^–1^ in the spectrum of the CEpCUR particles is a confirmation of the incorporation of curcumin in the particles.^51,52^ The ultraviolet–visible (UV–vis) spectroscopy analysis of the supernatant revealed a curcumin content of 0.73 mg remaining in the media after the fabrication of the CEpCUR particles and therefore an incorporation rate of about 82%, which validates the potential of these particles to encapsulate this hydrophobic model drug. This is in line with the results obtained for the incorporation of curcumin in particles obtained from other cellulose derivatives^53,54^ as reported in the study of Zamansky et al.^55^ in which ethyl cellulose particles entrapped around 80% of the curcumin used in the process. The presence of this model compound in the CEpCUR particles was further corroborated by their bright yellow color when compared with the white color observed for the pristine CEp counterparts, as depicted in Figure 2C.

Considering that these particles are aimed for the preparation of cell-laden biomaterials, the *in vitro* cytotoxic potential of CEp and CEpCUR was evaluated against HaCaT cells using the 3-(4,5-dimethylthiazolyl-2)-2,5-diphenyltetrazolium bromide reduction (MTT) assay. This cell line was selected envisioning the potential use of these bioinks for the 3D bioprinting of living structures for epidermal skin regeneration and topical drug delivery.^44,56,57^ The results summarized in Figure 2D show that the CEp particles had no cytotoxic effect after 24 h, with cell viabilities of 98.0 ± 3.4%, 104.8 ± 5.8%, and 82.7 ± 12.3% for concentrations of 1, 5 and 10% of particles, respectively. Similarly, CEpCUR particles also revealed no significant cytotoxic effect in the tested concentrations, with cell viabilities of 96.7 ± 8.4% (CEpCUR_1%), 88.9 ± 3.8% (CEpCUR_5%), and 83.3% ± 3.4% (CEpCUR_10%) after 24 h. Since all of the cell viability values obtained are well above the threshold of 70%, both particles (with and without curcumin) are considered noncytotoxic toward HaCaT cells in the investigated concentrations, as defined by the ISO 10993-5:2009, thus confirming their potential for biological applications like 3D bioprinting.^58^ Furthermore, the results are consonant with studies with other particles available in the literature, namely the works of Varshosaz et al.,^59^ using cellulose acetate butyrate particles loaded with nevirapine for HIV treatment (reporting viabilities above 80% against J774A1 cells), and of González et al.^60^ concerning the use of cellulose acetate phthalate/chitosan particles for the administration of captopril using HFF-1 cells.

### 3.2 Characterization of the Alginate Hydrogel-Based Inks

**Figure 3:**
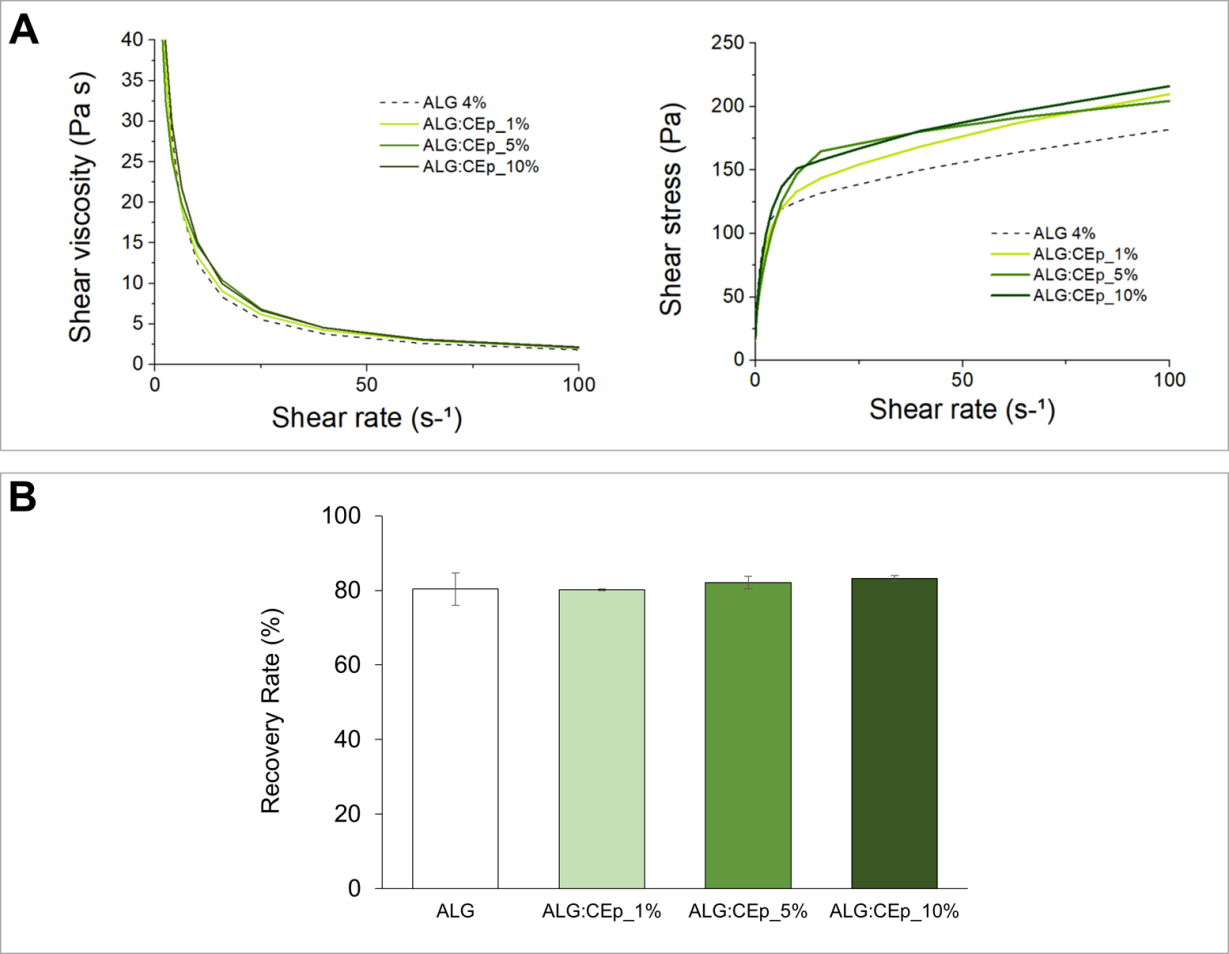
(A) Rheological evaluation (shear viscosity and shear stress as a function of shear rate) and (B) recovery rate (%) of the elastic modulus (*G*′) of ALG:CEp inks. Values are presented as the mean of three replicates.

The printability of hydrogel-based inks is greatly influenced by their specific rheological properties.^61^ Considering this, a thorough evaluation of the behavior of the composite hydrogels under stress is fundamental to predict their performance in the 3D printing process. First, the viscosity and shear stress of the ink formulations were assessed as a function of shear rate. As observed in Figure 3A, an increase in both parameters was observed with increasing amounts of CEp, indicating an improvement of the rheological properties of the alginate hydrogels with the addition of CEp. A similar phenomenon has already been described for other particles, as for example the study of Im et al*.*^24^ that reported an increase on the viscosity of pre-cross-linked alginate hydrogels with the addition of polydopamine particles, and Wu et al.^25^ reported similar results with cellulose nanocrystals.

Moreover, the significant decrease in shear viscosity of the inks with increasing shear rate proves that they have a shear-thinning behavior, a very relevant characteristic for 3D printing endeavors.^61^

The assessment of the recovery rate of the ALG:CEp inks was performed to evaluate the recovery of the rheological properties (namely, the *G*′ modulus) after intense stress, emulating the forces applied during the extrusion printing process. The data shown in Figure 3B revealed that all samples retain a high recovery rate, when compared with the blank alginate hydrogel (80.34 ± 4.28%), with values above 80% (80.17 ± 0.20%, 82.09 ± 1.67%, and 83.21 ± 0.73% for the ALG:CEp_1%, ALG:CEp_5%, and ALG:CEp_10%, respectively). Comparable recovery rates have also been described by Lan et al*.*^26^ for alginate hydrogels (81.6%) and by Zhang et al.^62^ for composite hydrogels of alginate, gelatin and graphene oxide (79.55%). Therefore, the presence of CEp did not compromise the capacity of the hydrogels to recover most of their original rheological properties after being submitted to the intense forces that mimic the extrusion process, allowing the hydrogels to retain their shape after printing.

**Figure 4:**
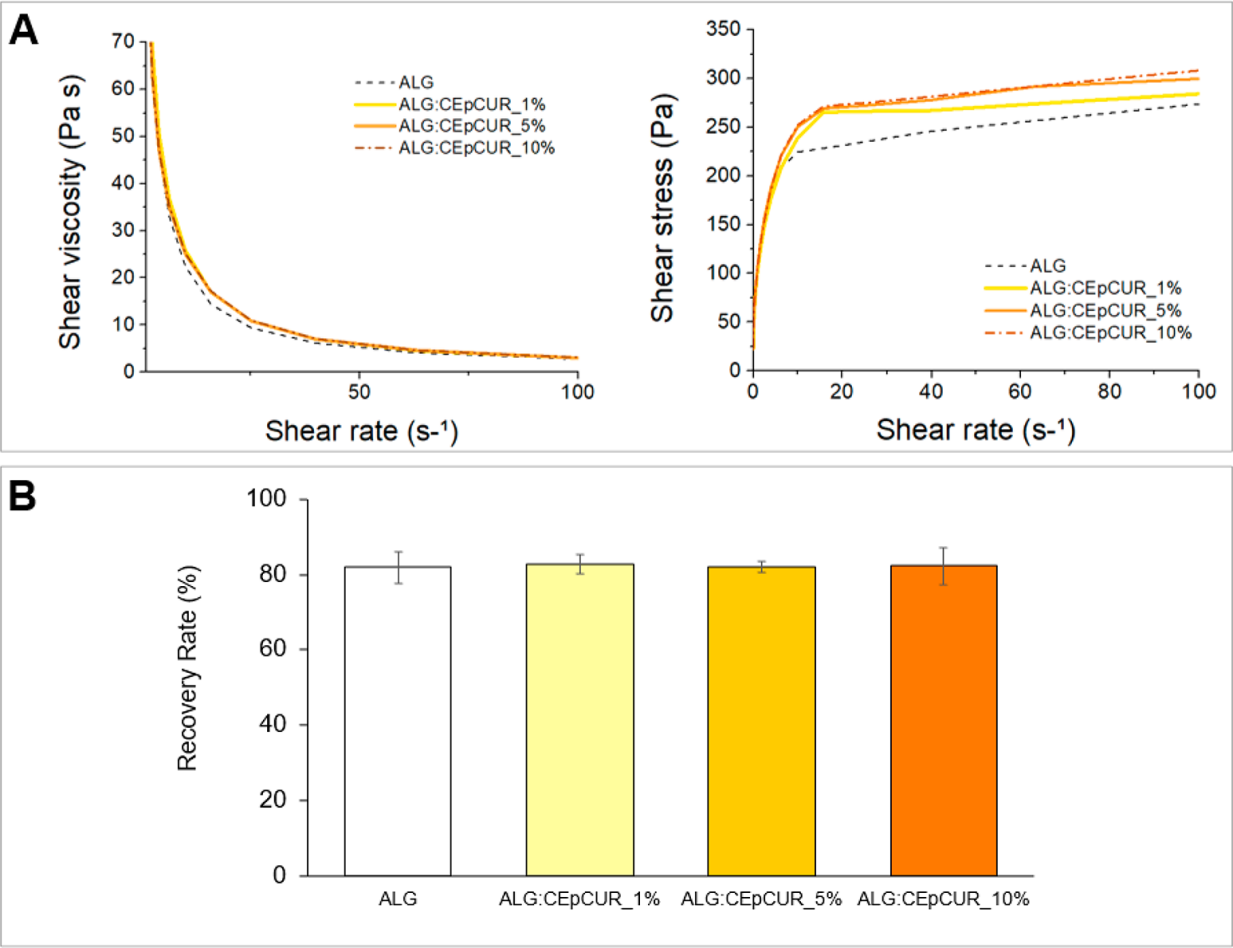
(A) Rheological evaluation (shear viscosity and shear stress as a function of shear rate) and; (B) Recovery rate (%) of the elastic modulus (*G*′) of ALG:CEp inks. Data are presented as the mean of three replicates.

Aiming to clarify the impact of the incorporation of curcumin in the properties of the inks, we subjected the ALG:CEpCUR hydrogel inks to the same rheological characterization described above for the ALG:CEp counterparts. The results shown in Figure 4 are analogous to those obtained for ALG:CEp inks. Once more, the notorious shear-thinning behavior observed for the pre-cross-linked ALG:CEp inks is also perceptible here, with an evident decrease in the viscosity with increasing shear rate (Figure 4A). Likewise, the recovery rates also remained unaltered by the incorporation of curcumin-loaded particles in the hydrogels (Figure 4B), with ALG:CEpCUR_1% recovering 81.92 ± 4.23%, ALG:CEpCUR_5% with 81.95 ± 1.52%, and ALG:CEpCUR_10% showing a 82.34 ± 4.90% recovery rate. All of these values are analogous to those obtained for ALG:CEp inks. Given this, the incorporation of curcumin does not impact the rheological properties of the composite inks, and the rheological properties described here for the alginate hydrogel-based inks with CEp or CEpCUR are considered appropriate for 3D bioprinting purposes.^61^

### 3.3 Characterization of the Fully Cross-Linked Hydrogels

Although the understanding of the rheological properties of the inks is essential to shed a light on their behavior during 3D extrusion-based bioprinting, the properties of the resulting fully cross-linked composite hydrogels are also of great importance, hinting at the potential of the final 3D constructs for posterior applications. Therefore, fully cross-linked hydrogels obtained from ALG, ALG:CEp, and ALG:CEpCUR inks were characterized in terms of their viscoelastic and mechanical properties, degradability, and *in vitro* cytotoxicity.

**Figure 5:**
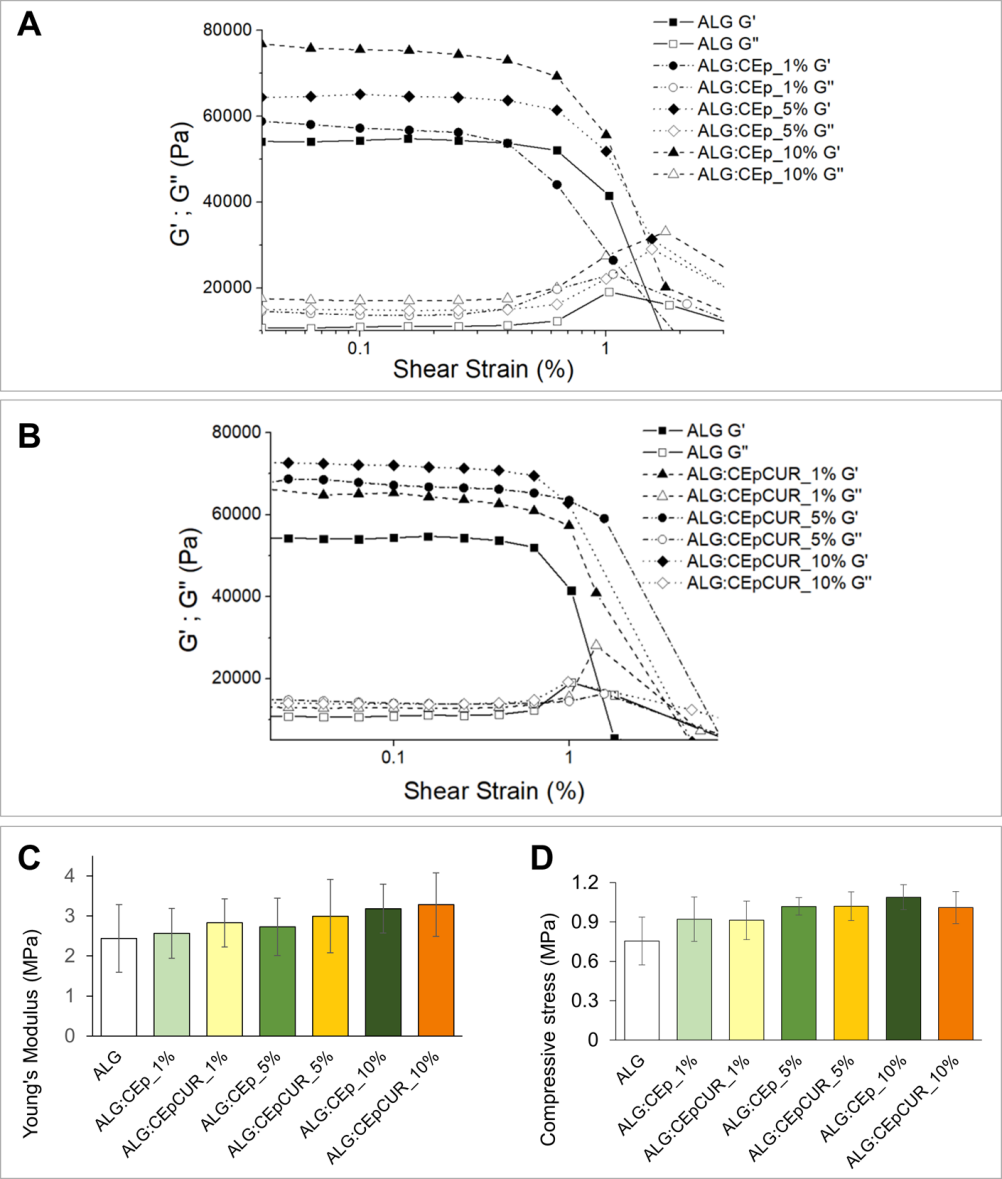
(A, B) Evaluation of the *G*′ and *G*″ moduli of the fully cross-linked hydrogels obtained from the (A) ALG:CEp or (B) ALG:CEpCUR inks; (C, D) mechanical properties of the ALG, ALG:CEp, and ALG:CEpCUR inks, namely (C) Young’s modulus and (D) compressive stress. Data are presented as the mean of five replicates, with no significant statistical differences.

The results of the evaluation of the elastic modulus (*G*′) and viscous modulus (*G*″) as a function of shear strain of the fully cross-linked hydrogels obtained from ALG, ALG:CEp and ALG:CEpCUR are shown in Figure 5A, B. As observable, the *G*′ is above the values of the *G*″ for all samples regardless of their compositions, indicating that all of the fully cross-linked hydrogels possess a solid-like and shape-supporting behavior that is desirable for 3D printing applications,^5^ similarly to other alginate hydrogels already described in the literature, like the alginate/gelatin/nanocellulose hydrogels developed by Han et al.^63^

Regarding their mechanical properties, all of the hydrogels were subjected to compression assays, and the results are shown in Figure 5C, D. As observed, the alginate hydrogel shows the lowest Young’s modulus value with 2.43 ± 0.84 MPa, and a slight increase is found with the rising concentration of particles. Nonetheless, this increase is not considered statistically significant. A similar phenomenon is witnessed for the compressive stress of the hydrogels at 80% strain (Figure 5D). Given this, the results from the mechanical compression assays confirm that the addition of CEp or CEpCUR to the alginate hydrogel matrix does not compromise the mechanical performance (namely Young’s Modulus and compressive stress) of the cross-linked hydrogels, hence preserving their potential for 3D bioprinting applications.

**Figure 6:**
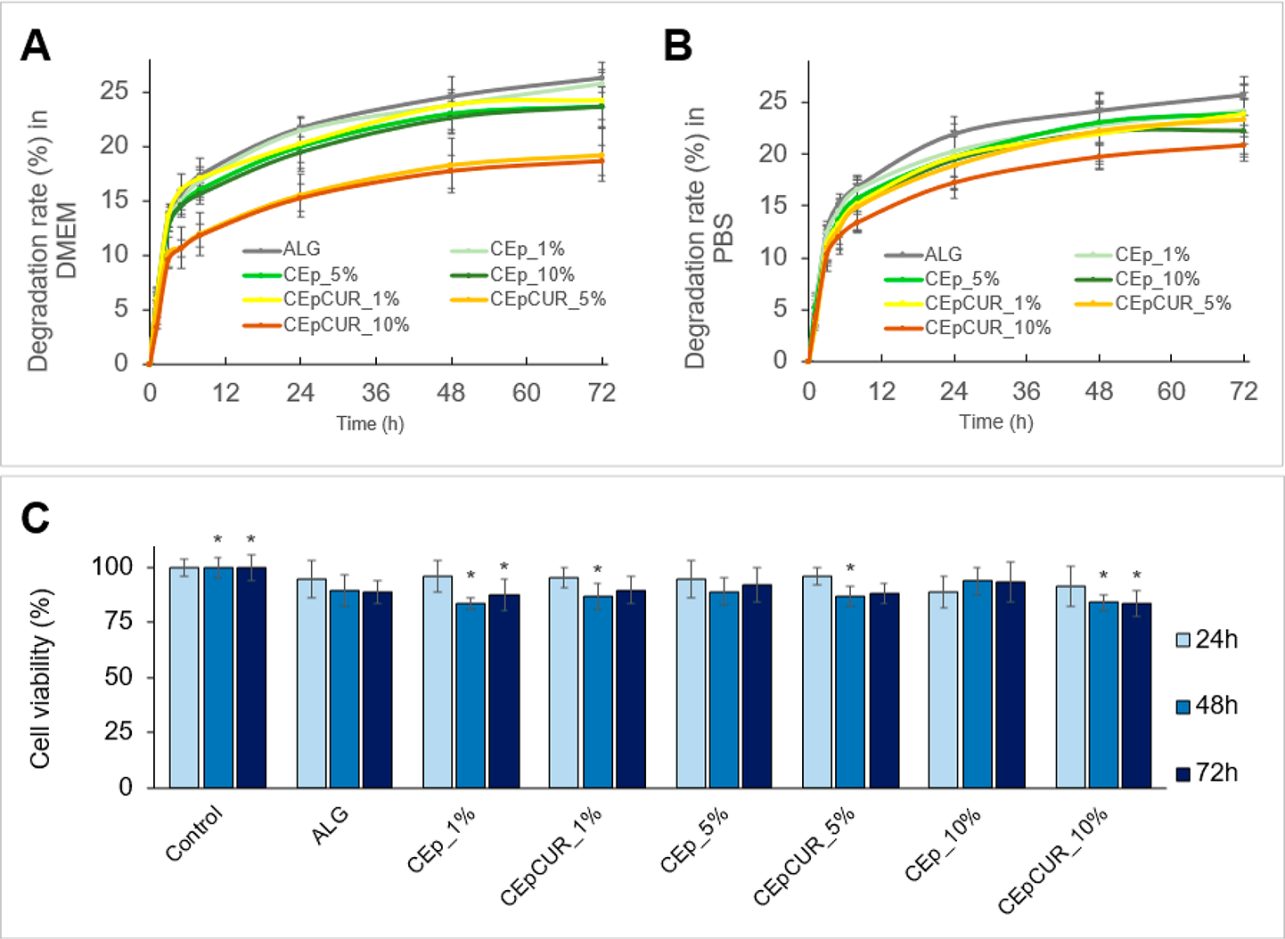
(A, B) Degradation rate (%) of the ALG, ALG:CEp, and ALG:CEpCUR inks in DMEM and PBS for 72 h at 37 °C; (C) evaluation of the cell viability of HaCaT cells after 24, 48, and 72 h of exposure to the alginate hydrogel, and the ALG:CEp and ALG:CEpCUR composite hydrogels. Presented values are the mean of six replicates (*p* < 0.05: *, relative to the comparison with the respective control).

The evaluation of the degradability of the hydrogels is also very important to understand the stability of the bioprinted constructs. Here, the degradation of the hydrogels was evaluated as their mass loss in different media (viz. cell-culture media (DMEM) and phosphate buffer saline (PBS, pH 7.4)) for 3 days at 37 °C. The results presented in Figure 6 show that the degradation of the alginate hydrogel in DMEM is faster in the first 12 h, reaching a degradation plateau of 26.3 ± 0.8% after 72 h. All of the composite hydrogel counterparts, however, show less pronounced degradation rates, indicating that the presence of CEp or CEpCUR in the alginate matrix affects the degradation of the ensuing hydrogels.

Interestingly, CEpCUR_5% and CEpCUR_10% hydrogels show the lowest degradation after 72 h, with values of 19.2 ± 2.4% and 18.7 ± 1.3%, respectively, noticeably below those of the curcumin-free samples. Curcumin is a molecule with very low water solubility, and so it may hamper the degradation of the alginate hydrogel matrix by limiting the entrance of water into the hydrogel matrix.^37^ A similar tendency is observed for the degradation of the hydrogels in PBS, with CEpCUR_10% reaching values of 20.9 ± 1.8% of degradation, while the ALG and ALG:CEp inks have higher degradation rates (25.6 ± 1.2% and 22.3 ± 2.9%, respectively). These values are lower than the results reported in other works concerning alginate hydrogels, like the study of Zidarič et al.^19^ using alginate and carboxymethyl cellulose (around 30% after 72 h). The higher degradation found by the authors is probably justified by the absence of CEp particles and curcumin.^19^

Another important parameter when developing materials for biomedical applications is their noncytotoxicity, as previously proved for the CEp particles. Given so, the cytotoxic potential of the fully cross-linked hydrogels against HaCaT cells was investigated (Figure 6C). All of the tested hydrogels revealed no cytotoxic impact against HaCaT cells for 24, 48, or 72 h, with cell viabilities above the 70% threshold. These results are in line with the well-known biocompatible nature of alginate^8,44^ and with the noncytotoxic effect of CEp and CEpCUR in the concentrations used here, as previously discussed. As such, this data set confirms the safety of the composite inks developed in the present work to be laden with HaCaT cells for 3D bioprinting applications.

### 3.4 3D Printing of Drug-Releasing Structures

**Figure 7:**
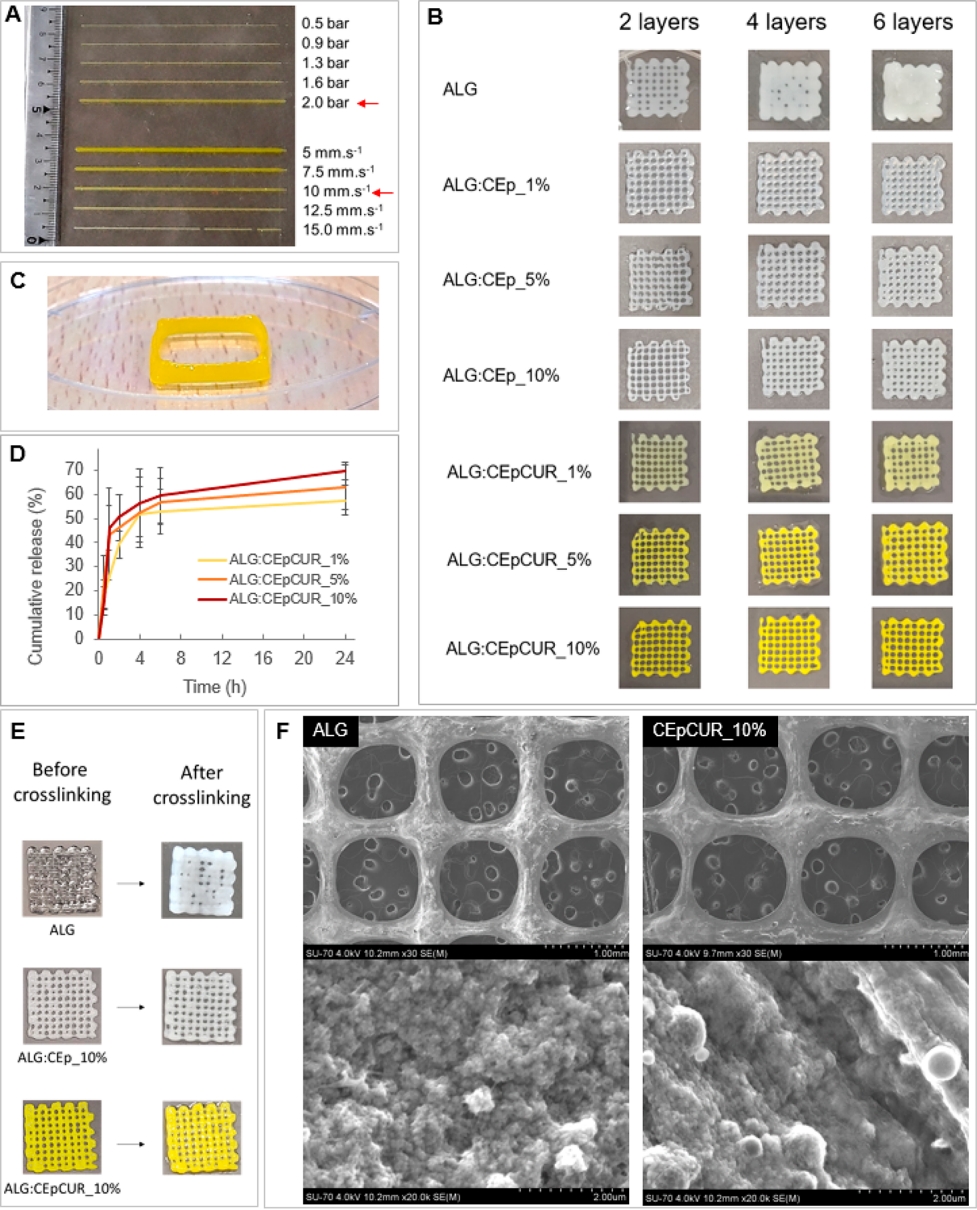
(A) Example of the optimization of the 3D printing conditions, namely, printing pressure (with fixed printing speed of 10 mm s^–1^) and printing speed (with fixed printing pressure of 2 bar) using the ALG:CEpCUR_10% ink; (B) 3D printed grid-like structures with different (2, 4, or 6) layers from ALG, ALG:CEp, and ALG:CEpCUR inks; (C) hollow 3D structure (20 mm × 20 mm) with 10 layers printed using the ALG:CEpCUR_10% ink; (D) cumulative release (%) of curcumin from grid-like structures obtained from the ALG:CEpCUR inks; (E) 3D printed grid-like structures with 6 layers before and after complete cross-linking with CaCl_2_; (F) SEM micrographs of grid-like structures obtained using ALG and ALG:CEpCUR_10% inks.

A detailed optimization of the 3D bioprinting process parameters is vital for the successful printing of complex constructs. Here, the optimization procedure was performed by first printing straight filaments of each of the composite inks. The results, exemplified in Figure 7A for the ALG:CEpCUR_10% ink, show that the inks containing CEp or CEpCUR could be successfully printed at 20 °C, using a nozzle with 0.25 mm inner diameter, a printing speed of 10 mm s^–1^ and a pressure of 2 bar.^26,44^ These conditions allowed the extrusion of a straight filament of ink with regular width without dispensing exaggerated amounts of the inks or the breaking of the strand.

Then, grid-like structures with multiple layers were 3D printed using the ALG, ALG:CEp and ALG:CEpCUR inks. The structures, represented in Figure 7B, clearly show that the use of ALG:CEpCUR inks results in constructs with the lively yellow color of curcumin, when compared with structures obtained exclusively from ALG or ALG:CEp, and that higher contents of CEpCUR lead to a more vibrant yellow tone. Furthermore, all of the constructs obtained by printing the composite hydrogel inks (ALG:CEp and ALG_CEpCUR) show better resolution, with a superior definition of the grid-like structure, when compared with the ones obtained from alginate, as seen for the 6-layered constructs before and after cross-linking (Figure 7E). In fact, the printability (*Pr*) of the ALG:CEpCUR bilayered constructs (0.9), is within the desired range for bioprinting applications^61^ (*Pr* = 0.9–1.0), and it is higher than that of the ALG counterpart (*Pr* = 0.8). This is certainly related with the improved rheological properties of the composite inks, as demonstrated before (viz. increased shear viscosity and shear stress), and comparable to the value obtained, for instance, on the study of Im et al.^24^ mentioned before, using a composite bioink of alginate, cellulose and polydopamine nanoparticles (*Pr* = 0.9).

Moreover, the SEM analysis of the 3D grid-like constructs obtained with the ALG:CEpCUR_10% ink shows that the composite hydrogels originate structures with the spherical CEpCUR particles embedded and on the surface of the hydrogel matrix (Figure 7F). These particles are homogeneously spread on the 3D constructs and remain spherical and intact even after the extrusion-based 3D printing process, indicating their potential to protect the cargo from external forces applied to the inks during this procedure. These particles are not present in the SEM micrographs of the ALG constructs. As a proof of concept, the construction of a bigger structure is exemplified in Figure 7C, where the ALG:CEpCUR_10% ink was used to build a hollow square of 20 mm × 20 mm. This 10-layered structure successfully maintained its shape, even in the absence of an inner supporting grid. Considering all of these results, CEp and CEpCUR are considered suitable additives for alginate-based inks, improving the dimensional stability of the 3D printed constructs.

The release mechanism of curcumin from the 3D printed grid-like structures obtained with the ALG:CEpCUR inks was evaluated in PBS at 37 °C, mimicking the physiological conditions. As shown in Figure 7D, the release of curcumin from ALG:CEpCUR_10% structures is characterized by an initial burst release in the first 4 h, followed by a plateau at around 8 h with a final cumulative release of 69.8 ± 3.7% after 24 h. A similar profile was observed for ALG:CEpCUR_5% and ALG:CEpCUR_1%, with cumulative releases of 63.1 ± 9.4% and 57.7 ± 6.4%, respectively. The curcumin release profile from the printed structures can be fitted to the Korsmeyer–Peppas model, in which *M*_*t*_/*M*_∞_ = *kt*^*n*^, (where *M*_*t*_ corresponds to the amount of curcumin released at time *t*, *M*_*∞*_ represents the amount of curcumin released at infinite time, *n* corresponds to the diffusion constant, and *k* is the kinetic constant).^64^ This model considers only the values when *M*_*t*_*/M*_*∞*_*<* 60%; given this, an *n* value below 0.5 (*n* = 0.131 for ALG:CEpCUR_10%, *n* = 0.118 for ALG:CEpCUR_5%, and *n* = 0.436 for ALG:CEpCUR_1%, *R*^2^ = 0.9953, 0.9649, and 0.9682) was obtained for these data, which is representative of a Fickian diffusion-controlled drug-release mechanism.^64,65^ Overall, these results confirm that the presence of CEpCUR in the alginate hydrogels grants the 3D printed constructs, apart from the improved printability and stability, with the ability to release active molecules. Moreover, this release mechanism is appropriate for tissue regeneration applications like wound healing, where a burst release in the first few hours could be beneficial for specific therapeutic options such as pain-relief or anti-inflammatory effects.^66^

### 3.5 3D Bioprinting of HaCaT Cells

The characterization of the inks, described in the previous sections, hints at the potential of these hydrogels for the successful 3D bioprinting of living cells. Nonetheless, the process of 3D bioprinting with cell-laden bioinks is often challenging and delicate. In fact, the cells are subjected to significant stress during this procedure, and the maintenance of high cell viability throughout the extrusion step and in the final construct is the ultimate test to the adequacy of a newly developed bioink.

**Figure 8:**
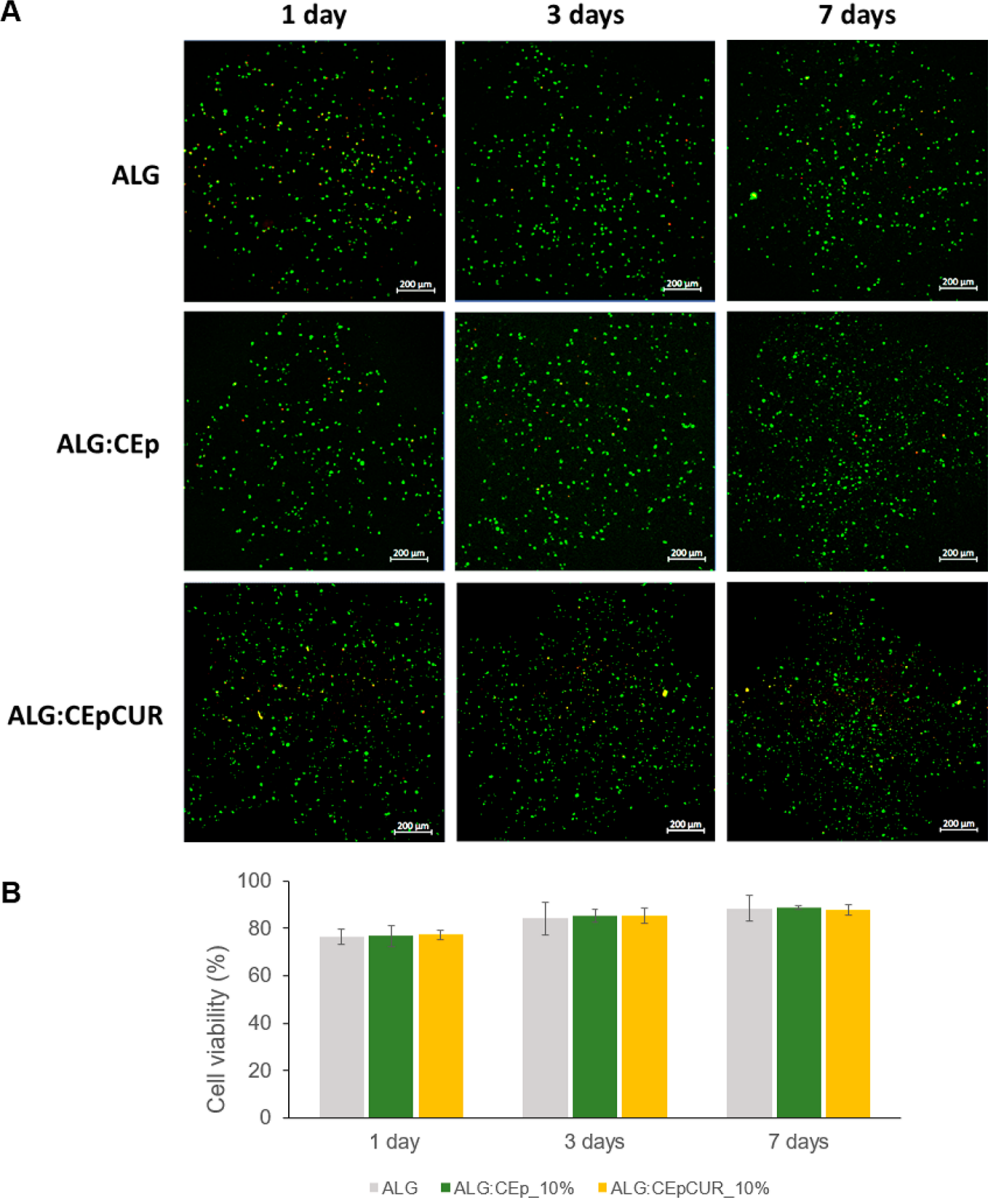
Evaluation of the cell viability on 3D bioprinted constructs using ALG, ALG:CEp_10% and ALG:CEpCUR_10% bioinks. (A) LIVE/DEAD fluorescence micrographs of the HaCaT cells encapsulated in the constructs after 1, 3, and 7 days post-bioprinting. Live and dead cells are marked in green and red, respectively. (B) Results of the determination of cell viability (%) at days 1, 3, and 7 post-bioprinting. Data are presented as the mean of three measurements with no significant statistical differences.

In this work, HaCaT cells were loaded into the alginate hydrogel with higher contents of particles (viz. ALG:CEp_10% and ALG:CEpCUR_10%), taking into account the good results of the cytotoxicity assays described above and the enhanced properties of these ink formulations (viz. rheological features, degradability, and printability). The pristine ALG hydrogel was similarly loaded with living cells for comparison purposes. Then, grid-like structures were 3D bioprinted with the bioinks, using the previously optimized parameters. The results of the evaluation of cell viability in the bioprinted constructs after 1, 3, and 7 days post-bioprinting, performed by LIVE/DEAD assay, are depicted in Figure 8.

The fluorescence micrographs show that HaCaT cells are homogeneously distributed in all constructs, with a clear prevalence of living cells (marked in green) over dead cells (in red), regardless of the time point. Specifically, ALG:CEp_10% and ALG:CEpCUR_10% maintain good cell viabilities 1 day after the bioprinting procedure, with values of 76.8 ± 4.3% and 77.3 ± 1.9%, respectively. These values are very similar to the results obtained for the ALG hydrogel (76.6 ± 3.1%), proving that the presence of CEp and CEpCUR in the matrix does not compromise the successful 3D bioprinting of HaCaT cells, maintaining high cell viabilities. Moreover, these viabilities are preserved after 3 days (with ALG showing 84.1 ± 6.8%, ALG:CEp_10% with 85.4 ± 2.6%, and ALG:CEpCUR_10% with 85.3 ± 3.2% of cell viability), slightly increasing until the last time point at 7 days, where the ALG bioink reveals 88.4 ± 5.3% cell viability, and ALG:CEp_10% and ALG:CEpCUR_10% bioinks show 88.9 ± 0.9% and 87.8 ± 2.3% cell viabilities, respectively. Other works concerning the 3D bioprinting of alginate-based hydrogels describe similar results, including the work of Lan et al.^26^ that used TEMPO-oxidized cellulose/alginate hydrogels for the bioprinting of human meniscus fibrochondrocytes, and the study of Huang and colleagues^20^ where fibroblasts showed cell viabilities above 85% 2 days after bioprinting in a gelatin/sodium alginate/carboxymethyl chitosan bioink. Regarding the 3D bioprinting of HaCaT cells, our team has shown comparable outcomes using other bioink formulations, like the work of Teixeira et al.,^44^ about alginate hydrogels with lysozyme nanofibrils, that showed viabilities of nearly 88% after 7 days; and the study by Lameirinhas et al.^67^ regarding a bioink composed of gellan gum and cellulose nanofibers, with HaCaT cell viabilities of 90 ± 3%.^67^

These results confirm that the composite alginate hydrogel-based bioinks with cellulose ester-based particles are adequate for 3D extrusion bioprinting of living constructs with HaCaT cells, maintaining a high cell viability up to 7 days after the 3D bioprinting procedure. Moreover, the prior inclusion of a molecule of interest (i.e., a model drug) in the cellulose particles enables the creation of living constructs with drug-releasing ability, which constitutes an innovative approach for biomedical applications such as wound healing. In fact, the antioxidant and anti-inflammatory properties of curcumin have been explored before for wound healing applications,^68,69^ including in the treatment of burns and diabetic ulcers, and these therapeutic effects are also being explored for the engineering of cardiac, musculoskeletal and cartilage tissues, corroborating the relevance of the inclusion of this molecule in the ink formulations.^70^

## 4 Conclusion

The present work reports for the first time the use of spherical particles of cellulose esters as additives for alginate hydrogel-based bioinks using them simultaneously as strengthening agents and as carriers for drugs or bioactive compounds. The incorporation of CEpCUR (in 1, 5, or 10 wt %) in the alginate hydrogels (4% w/v) enhanced their rheological properties (i.e., by increasing shear viscosity and shear rate), the printability of the formulations (*Pr* = 0.9) and resulted in constructs with a higher definition of the predefined structure. ALG:CEpCUR inks originated yellow-colored fully cross-linked hydrogels with lower degradation rates (19.2 ± 2.4% and 18.7 ± 1.3% for CEpCUR_5% and CEpCUR_10%, respectively) when compared with the pristine ALG counterpart (26.3 ± 0.8%), as evaluated for 3 days at 37 °C. The 3D bioprinted constructs successfully release curcumin into the media, as evidenced by the release of 69.8 ± 3.7% of the drug after 24 h. Furthermore, these inks present no cytotoxic potential against HaCaT cells for up to 72 h, representing an adequate environment for cells to thrive, as observed by the high cell viabilities (nearly 90%) 7 days after bioprinting. Given so, this work demonstrates the possibility of creating functional composite alginate-based bioinks that act simultaneously as the support matrix for cells in the 3D bioprinting procedure and also as suitable vehicles for the delivery of drugs and other bioactive molecules, originating living tissues with drug-release capabilities. These living structures constitute a new approach for future biomedical applications, including wound healing.

## Abbreviations

ALG: alginate
CEp: cellulose ester-based particles
CEpCUR: curcumin-loaded cellulose ester-based particles
CUR: curcumin
dECM: decellularized extracellular matrix
DMEM: Dulbecco’s modified Eagle’s medium
DMSO: dimethyl sulfoxide
FBS: fetal bovine serum
FTIR-ATR: Fourier transform infrared-attenuated total reflection spectroscopy
MTT: 3-(4,5-dimethylthiazolyl-2)-2,5-diphenyltetrazolium bromide
PBS: phosphate buffer saline
SEM: scanning electron microscopy
UV–vis: ultraviolet–visible spectroscopy
